# Cell-Sorting at the A/P Boundary in the *Drosophila* Wing Primordium: A Computational Model to Consolidate Observed Non-Local Effects of Hh Signaling

**DOI:** 10.1371/journal.pcbi.1002025

**Published:** 2011-04-07

**Authors:** Sabine Schilling, Maria Willecke, Tinri Aegerter-Wilmsen, Olaf A. Cirpka, Konrad Basler, Christian von Mering

**Affiliations:** 1 Institute of Molecular Life Sciences, University of Zurich, Zurich, Switzerland; 2 Swiss Institute of Bioinformatics, University of Zurich, Zurich, Switzerland; 3 Center for Applied Geoscience, University of Tuebingen, Tuebingen, Germany; Princeton University, United States of America

## Abstract

Non-intermingling, adjacent populations of cells define compartment boundaries; such boundaries are often essential for the positioning and the maintenance of tissue-organizers during growth. In the developing wing primordium of *Drosophila melanogaster*, signaling by the secreted protein Hedgehog (Hh) is required for compartment boundary maintenance. However, the precise mechanism of Hh input remains poorly understood. Here, we combine experimental observations of perturbed Hh signaling with computer simulations of cellular behavior, and connect physical properties of cells to their Hh signaling status. We find that experimental disruption of Hh signaling has observable effects on cell sorting surprisingly far from the compartment boundary, which is in contrast to a previous model that confines Hh influence to the compartment boundary itself. We have recapitulated our experimental observations by simulations of Hh diffusion and transduction coupled to mechanical tension along cell-to-cell contact surfaces. Intriguingly, the best results were obtained under the assumption that Hh signaling cannot alter the overall tension force of the cell, but will merely re-distribute it locally inside the cell, relative to the signaling status of neighboring cells. Our results suggest a scenario in which homotypic interactions of a putative Hh target molecule at the cell surface are converted into a mechanical force. Such a scenario could explain why the mechanical output of Hh signaling appears to be confined to the compartment boundary, despite the longer range of the Hh molecule itself. Our study is the first to couple a cellular vertex model describing mechanical properties of cells in a growing tissue, to an explicit model of an entire signaling pathway, including a freely diffusible component. We discuss potential applications and challenges of such an approach.

## Author Summary

In developing animal tissues, cells can often re-arrange locally and mix relatively freely. However, in some stereotypic and crucially important instances during body development, cells will strictly not intermingle, and instead form sharp boundaries along which they will sort out from each other. This mechanism helps organisms to establish signaling centers and to maintain distinct cellular identities. Often, cells at such boundaries will remain in close physical contact and are morphologically alike. Thus, the boundary itself can be difficult to observe unless the expression status of specific marker genes is monitored experimentally. How are these ‘compartment boundaries’ established? Here we devise a computational model that aims to describe one such boundary in a well-studied animal tissue: the developing wing primordium of *Drosophila melanogaster*. We model the production, diffusion and local sensing of an essential signaling molecule, the *Hedgehog* protein. We reveal one possible mechanism by which Hedgehog sensing can influence the mechanical properties of cells, and compare the simulated outcome to observations in experimentally perturbed, actual wing discs. Our relatively simple model suffices to establish a straight and stable compartment boundary.

## Introduction

During embryonic development of complex multicellular organisms, spatial reference points need to be established within tissues. These are often formed by specialized groups of cells that are capable of signaling to neighboring cells. Such signaling centers define coordinate systems along which newly arising cells can orient themselves and make crucial decisions regarding proliferation, differentiation or migration [1], [2], [3], [4], [5], [6]. Because of their pervasive importance, tissue-organizing centers need to be precisely controlled – both spatially and temporally, as well as with respect to their signaling amplitude.

One possible mechanism for spatial control of tissue organizers is to restrict the movement of cells at fixed boundary positions. This phenomenon is indeed observed, and it involves the separation of groups of cells that have already been spatially instructed to assume distinct identities, for example at segment- or parasegment-boundaries. Akin to water in oil, the two cell populations are seen to establish and maintain a relatively straight interface to each other, effectively minimizing their contact area. The minimizing force is assumed to help stabilize the interface against random perturbations that may arise from cell divisions or from arbitrary cell movements; thus, any organizing activity that is associated with the interface is likewise stabilized. How is this separation, or ‘sorting’, of cells of distinct identities achieved? One line of work attributes this to differential cell adhesion [7], [8]: cell populations might develop distinct adhesive properties; these affinity differences would then allow them to sort out from one another. Another line of reasoning is based on Differential Interfacial Tension (DIT) [9], [10]: this hypothesis suggests that cells might actively constrict surfaces that are in contact with neighboring cells, depending on the cellular identity of neighbors and/or depending on signaling events. Both mechanisms would ultimately lead to physical forces that would help keep the cell populations apart.

**Figure 1:**
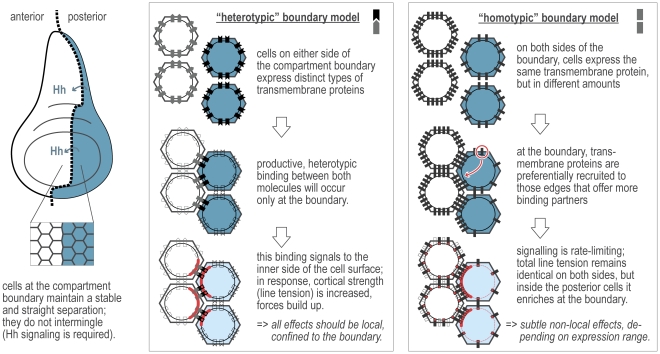
Two possible mechanisms for cell sorting in Drosophila wing discs. In the wing imaginal disc, cells of two distinct identities form the posterior and anterior halves, respectively. The demarcation line between them has been termed ‘compartment boundary’. The secreted signaling protein Hedgehog is emanating from the posterior section, and cells respond to this signal in the anterior section; this response is needed to maintain a well-defined separation between the two compartments. In the boxes to the right, two alternative scenarios for the molecular events leading to boundary formation are outlined.

The developing wing primordium of *Drosophila* (‘wing disc’) is particularly well suited to study boundary formation (Figure 1). It is not required for larval viability, can be manipulated experimentally through an advanced genetic toolkit, and has been well characterized. The disc contains a compartment boundary that separates anterior from posterior cells; this boundary is inherited from specification events occurring early in the embryo. The initial embryonic events that give rise to the boundary involve mutual signaling between stripes of cells, mediated by an extensively studied network of genes (the ‘segment polarity network’ [11], [12], [13], [14]). Once established, the cellular identities on both sides of these boundaries are stable throughout larval development and well into adult life. The compartment boundary in the disc is strictly respected by all cells, even when cells on one side of the boundary are artificially provided with a competitive growth advantage over cells on the other side of the boundary [15]. The wing disc itself is a simple, flat, epithelial sheet, and the orientations of cell divisions appear largely random [16]. Genetic analysis and computational modeling of this tissue is simplified by the fact that daughter cells arising from cell divisions usually remain in physical contact and do not migrate away from each other. This has been shown experimentally by tracing descendents of single cells; in most cases such a ‘clone’ of offspring cells will form a coherent patch of connected cells. This behavior suggests that the complicated processes of cell intercalation and migration can be neglected, to a first approximation, when studying boundary maintenance in this tissue.

**Figure 2:**
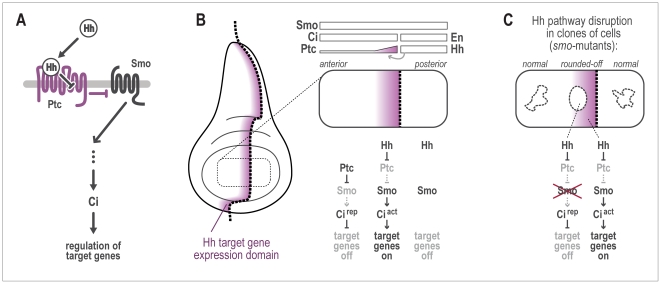
Summary of Hh signaling in the wing disc. A) The diffusible signaling protein Hh exerts its function by binding to its receptor Ptc. This relieves the transmembrane protein Smo from inhibition by Ptc. Smo then signals to the cytoplasm, where the signal is eventually relayed to the transcription factor Cubitus interruptus (Ci). Target genes under the control of Ci thus respond to Hh signaling. B) Expression domains of genes important in the process. Engrailed (en) and ci mark the two compartments. Hh is produced under the direction of en, and then diffuses freely. Smo is expressed throughout the disc. Ci is needed to activate or repress target genes in response to Hh signaling. Ci is not present in the posterior half, hence Hh target genes cannot be activated there. Immediately at the boundary, target genes exhibit the largest difference in expression. C) When small groups of cells are made mutant for the *smo* gene, they can no longer activate target genes. When such clones are located within the target gene stripe, they are known to round off and to tend to minimize contact with neighboring cells.

Working with such wing discs, a recent, seminal study has begun to shed light on possible boundary formation mechanisms [16] (see also ref [17]). The authors have directly demonstrated an increased mechanical tension at cell-to-cell interfaces located immediately at the boundary, using laser ablation experiments. Subsequent computer simulations then revealed that collectively such local forces are sufficient to maintain a stable compartment boundary. These results are intriguing, but they raise a number of new questions: Boundary formation in the wing disc is known to depend on the secreted and diffusible signaling protein Hedgehog (Hh), which is produced by posterior cells and specifically sensed and transduced by anterior cells [18], [19] (Figure 2). If diffusible Hh indeed somehow influences mechanical tension, what conditions must then be met to ensure a well-defined boundary? So far, all known transcriptional responses of Hh signaling are occurring several cell-diameters wide into the responding tissue. How is the response in this case restricted to the immediate boundary region? Furthermore, experimental suppression of Hh signaling has been shown to lead to ectopic boundary formation distant from the actual boundary [20]. Does this mean that the influence of Hh signaling does extend beyond the actual boundary, and if so, why does this not have a noticeable consequence in the wild type situation?

Here, we propose a mechanistic model that can generate a localized outcome of Hh signaling with respect to physical forces and mechanical properties, despite a longer range of the molecular response in terms of target gene expression. Furthermore, we estimate the distance from the boundary, up to which Hh signaling may be able ‘prime’ cells for boundary formation; this distance is inferred using both experimental results as well as modeling results, and we estimate it to be at least 10 cell diameters. We approach the problem by first formulating an explicit, two-dimensional model of Hh production, diffusion and transduction, and by then coupling this setup to a physical model of the growing tissue. In our modeling approach, cells and their contact surfaces are described as a graph of connected vertexes. Our model essentially follows the Differential Interface Tension hypothesis; it is a modified version of a model that has been previously established for the very same tissue [21]. We observe good compartment boundary formation over a range of simulation parameters, and the modeling outcomes agree qualitatively with experimental perturbations specifically performed for this study.

## Results/Discussion

In principle, at least two distinct molecular scenarios could explain the local generation of tensile forces at the boundary (Figure 1). In the first scenario (ref [16]), two different cell-surface molecules would form a heterotypic interaction at the boundary; their expression would essentially be under the control of the anterior or posterior “identities” of cells on either side of the boundary. The heterotypic interaction of these two molecules would be sensed locally at the cell-interaction interfaces, which would then respond by generating increased physical tension. This is a simple and attractive model, but it is not straightforward to consolidate with the known requirement, on the anterior side, for reception and transduction of the Hh signal. Loss of Hh transduction can generate ectopic boundaries in the anterior compartment ([20], this study), but it is generally not presumed that such loss of Hh signal will change the identity of anterior cells into that of posterior cells: the expression status of the selector genes engrailed and ci is not affected by Hh signaling. Thus, if the cell-identity seems unchanged, then both of the putative cell-surface molecules required for this model would have to be under Hh control: one as a direct molecular target, and one as an “inverse” molecular target (i.e., de-repressed upon the loss of Hh signal). Only in such a setup would loss of Hh transduction lead to ectopic boundary formation within the anterior compartment. However, target gene de-repression upon loss of Hh signal has to our knowledge never been reported for any known Hh target gene, and it would likely require further, more complicated indirect signaling mechanisms. Alternatively, one might imagine that one of the two molecules was expressed ubiquitously throughout the tissue, and only the other molecule would be a target of Hh signaling. However, in such a scenario heterotypic binding would occur throughout the entire Hh target gene expression domain; increased tension would thus not be restricted to the immediate compartment boundary only.

We therefore propose an alternative, somewhat more parsimonious scenario (Figure 1): The increased tensile forces at the boundary would be the consequence of a single cell-surface molecule, which would be a simple, direct molecular target gene of Hh signaling. This molecule would be able to transmit a signal to the inside of the cell, but only upon its activation by a homotypic interaction with molecules of the same type from a neighboring cell. Crucially, as discussed in more detail below, this signal and its conversion into mechanical tension would have to be rate-limited: relative tension would be highest at the section of the cell where most of the molecule has been activated, but the overall tension per cell would be constant (i.e. independent of the absolute amount of activated cell-surface molecule).

**Figure 3:**
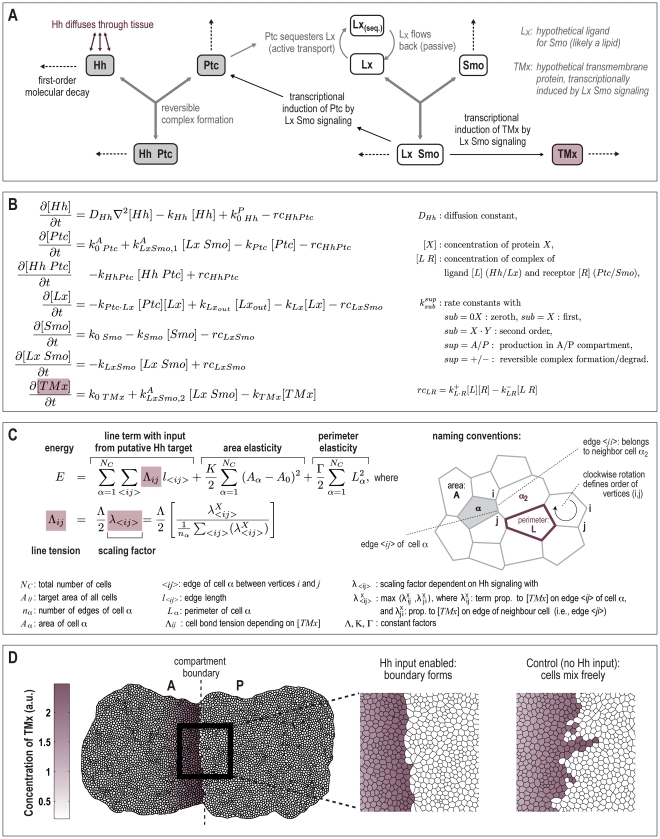
The modeling strategy. A) Mechanistic modeling of Hh signal transduction. Only events in the anterior compartment are shown. Dotted arrows denote the decay of either single molecules or complexes of molecules; the reversible formation of complexes is denoted by groups of three grey arrows. B) Differential equations describing the Hedgehog pathway. The mechanistic modeling depicted in A) is translated into a set of differential equations. This equation system is solved numerically for each cell after each growth step. C) Mechanical energy function describing cell shapes. Similar to ref. [21], we describe the tissue as a two-dimensional mesh of cells, whereby edges between vertices denote interaction interfaces between neighboring cells. Stable network configurations are defined by local minima of the energy function describing the separate contributions from the line tension between two vertices, each cell's area elasticity and the elasticity of each cell's perimeter [21], [37]. However, the term describing line tension (i.e. the energy ‘stored’ in a given edge) has been modified to include an additional scaling factor 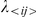, which depends on the ratio of the concentrations of “TMx” in the two cells sharing the edge *<ij>*. A detailed derivation of the scaling factor is given in Figure S3. D) Outcome of a simulation run. Simulations were started from 220 cells, growing up to 6000 cells. Left: Concentration gradient of the transmembrane protein TMx. The inset shows a magnified view of cells at the compartment boundary. In the control, the TMx input into the energy function has been disabled; note that in this case the boundary is quite irregular, due to disturbances by random cell division events. Videos of typical simulation runs for both control and experiment are provided in the Supplemental Information of this article (Videos S1 and S2).

To accurately take into account the role of Hh signaling in the disc (Figure 2), we first devised a formal model for the production, diffusion and transduction of Hh in this two-dimensional tissue. The model (Figure 3) includes the Hh receptor Patched (Ptc), as well as the essential downstream signaling component Smoothened (Smo), together with an unknown, putative co-factor of Smo; this co-factor is not further specified but has been speculated to be a lipid [22], [23]. The Hh protein is allowed to freely diffuse throughout the tissue, following its production in posterior cells. For each individual cell within the tissue, we compute the concentrations of the modeled entities as they develop in time by numerically solving a set of partial differential equations (Figure 3B). Apart from known or suspected players in Hh signaling, we implement an additional, putative target gene of Hh signaling, which we term “TMx”. Unlike Ptc, this gene is presumed to play no active role in the signaling pathway itself, instead it is a downstream target of the pathway and does not feed back into the sending or receiving of the Hh signal. We assume this gene to have the simplest possible connection to Hh signaling, namely a production term proportional to the amount of active Smo molecule in anterior cells. We further assume that the product of this gene has a function in regulating cortical tension at the inner surface of cells. We do not specify the molecular mechanism by which it regulates tension, but one could for example envisage TMx being a transmembrane protein whose intracellular domain recruits or otherwise influences cortical actin filaments [24]. Since TMx is modeled as a Hh target gene, it provides a way to connect transcriptional Hh responses to physical forces acting on cell shapes (Figure 3C). Our model is based on three central assumptions with regard to TMx: first, that it would increase cortical tension only in response to homotypic activation, i.e. upon binding another TMx molecule presented on the surface of a neighboring cell. Second, that it cannot increase the *overall* propensity of the cell for exacting cortical forces, but instead merely re-distributes cortical tension factors among the various interfaces that a given cell has with its neighbors. Again, we do not specify why this might be the case, but one could envisage a dynamic equilibrium of cytoskeleton filament deposition, and removal, at the cortex. In such a situation, each section of the cellular surface competes with all other sections within the same cell for the build-up of cytoskeleton material, and activated TMx might simply tip the balance towards deposition, locally. Lastly, the TMx molecule itself (while initially expressed isotropically) would enrich at cell surfaces at which it is activated by homotypic binding, perhaps because it is stabilized or preferentially re-deposited there. Thus, the overall effect of TMx would be that it changes the relative strength of contractile forces at each individual cell/cell contact segment; we model this as scaling factor within the line tension term of the physical energy function (Figure 3C).

For our implementation of the full model, a challenge was to accurately compute the two-dimensional diffusion of the Hh protein on a geometry that is itself constantly changing. We achieve this by alternating the mechanical relaxation/growth computations with an explicit diffusion of Hh on finite volumes established by the shapes of the cells (see Supplemental Material, Text S1). It should be noted that our model does not address questions related to overall regulation of tissue growth or to the determination of final organ size (nor does it address issues of correct developmental timing). Detailed models for growth control and mechanical forces affecting the tissue as a whole have been developed already [25], [26], [27], but they do not need to be applied here because our readouts are local, and because we stop the simulations well before the tissue would normally cease growing.

**Figure 4:**
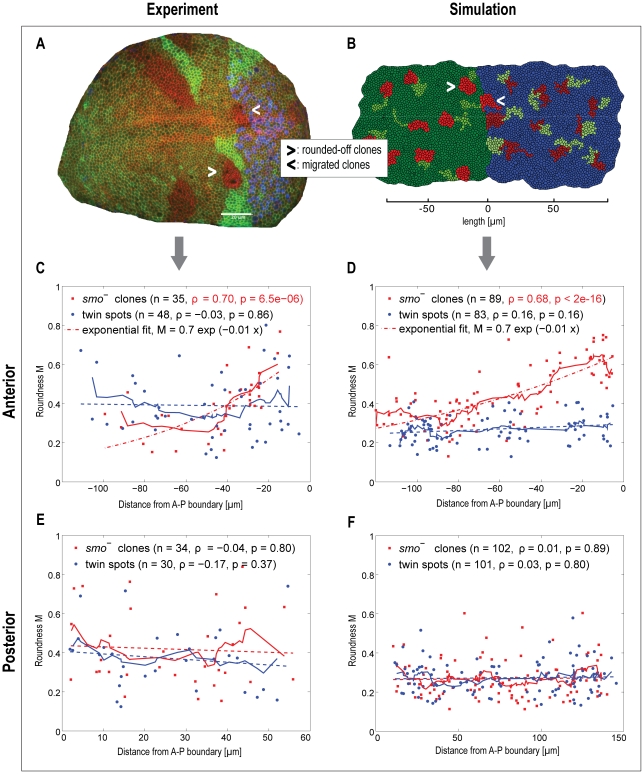
Experimental and simulated disruptions of Hedgehog signaling. The disruption of the crucial transduction protein Smo is an important assay revealing the functional roles of Hh signaling. A) Confocal microscopy image of a wing pouch with clones of cells mutant for *smo*. Posterior cells are marked in blue (via a *hh*-lacZ enhancer trap construct), cell outlines are marked in red (cadherin staining), and loss of *smo* is indicated by loss of GFP staining (i.e. absence of green color). Most clones are accompanied by a twin-spot (having two functional *smo* genes; bright green). Notice how an anterior clone close to the boundary has a ‘rounded’ appearance and seems to minimize contact with neighboring cells (marked by a white arrow). Another clone of anterior origin (i.e. absence of blue staining) happened to originate in immediate contact with the boundary. It has migrated into posterior territory, lost its roundness, and its leftward edge now constitutes a new boundary interface. B) Simulated wing pouch with clones of cells mutant for *smo* (marked in red) and their corresponding twin spots (marked in bright green). Simulations reproduce the experimentally observed ‘rounded’ appearance of clones in the boundary region of the anterior compartment as well as the migration of anterior clones close to the compartment boundary into the posterior compartment (see also Video S3). C) – F) Roundness of clones is used as an indirect measure of the strength of ectopic boundary formation. In both experiments (C) and simulation (D), *smo^−^* clones in the anterior compartment show a highly significant trending for decreasing roundness away from the boundary. In both cases, roundness is above background levels for at least 20 µ*m* (equivalent to at least 10 cells). In controls – i.e. posterior compartment clones, or wild-type twin spots – no significant trend is observed. For automatic image processing in C) and D), clones were required to be located at a minimum distance of 5 *µ* *m* from boundary, thus excluding clones migrating from anterior to posterior compartment in the analysis of roundness. C)–F) Straight lines: Moving average of the clonal (red) and twin spot (blue) shape distributions; Blue dashed lines: Linear fit of twin spot shapes vs. distance. E)–F): Red dashed lines: Linear fit of clonal shapes vs. distance in the posterior compartment.

Having specified the model, we next set out to parameterize it. Experimentally quantified data regarding the various kinetic parameters in Hh signaling are difficult to obtain and are at present quite sparse. We therefore focused our parameter exploration and validation on the modeled *shapes* of the various concentration gradients in the tissue (rather than on the absolute molecular concentrations); these shapes are already much better known, mainly from antibody staining experiments. For simplification, we performed parameter exploration in one dimension only, by projecting molecular concentration gradients along an anterior-posterior transect of the tissue (Figure S1). The Ptc protein in particular served as a guide for our manual parameter optimization – it is itself a target gene of Hh, and its expression and activity gradients are understood comparatively well [28]. As is shown in Figure S1, our model resulted in the characteristic up-regulation of Ptc in a small stripe of cells anterior to the boundary. Remarkably, the Ptc protein concentration gradient shows an approximately sigmoidal shape when projected along the antero-posterior axis, with highest values close to the boundary; this is not specified in the model as such, but instead follows naturally from the wiring of the pathway, with Ptc being both the receptor and a direct target gene of Hh. Because the parameter space of our model is fairly large, and each simulation run takes several CPU hours, a fully systematic scan of the possible parameters is difficult. Instead, we explored the parameter space manually. Thus, our parameter set should and will be updated as experimental data on concentrations and kinetic constants become available; any updates will again have to reproduce the known shapes of concentration gradients in the tissue. Initial test runs of our model revealed that several parameter sets resulted in the formation of a stable lineage boundary at the anterior/posterior interface (see for example Figure 3D and Figure 4). The resulting overall tissue-shapes often revealed a small constriction of the tissue margins at the position of the boundary, suggesting that the boundary exerts long-range mechanical forces on the tissue as a whole, as might be expected (Figure 3D).

**Figure 5:**
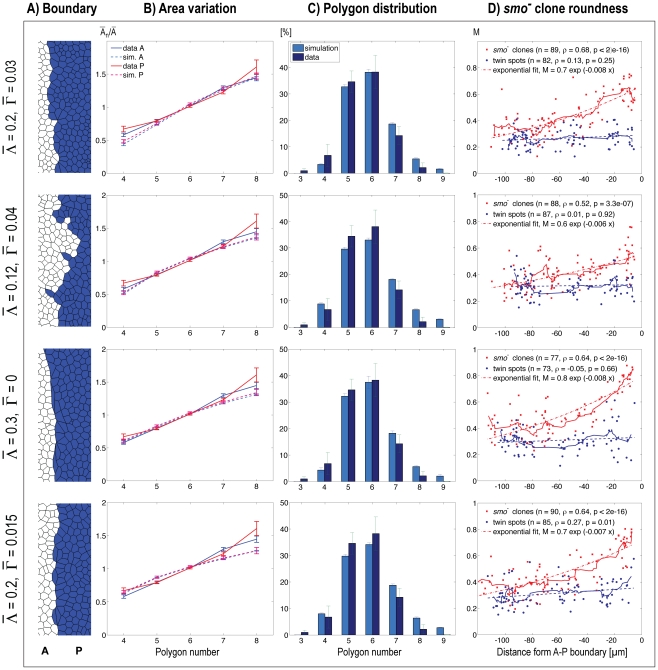
Parameter dependency. Different parameter choices for the energy function are observed to lead to compartment boundaries of varying stringency and straightness, but they will also influence descriptive statistics for cell and clonal shape distributions. The settings in the first row demonstrate the best overall fit to experimental data generated here and in ref [16], [21]. For each parameter set, we ran the simulations 10 times without the insertion of *smo* mutant cells (columns B–C), and 10 times with mutant cell insertions (column D). A) Zoom (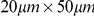) into the boundary region of a typical simulated wing pouch. B) Average apical cross-section areas of *n*-sided cells as a function of the polygon number *n*. Areas are normalized to the average apical cross - section area 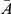 of each disc. C) Distribution of average polygon numbers. B) and C): Mean, and standard error of the mean (SEM), are shown for data and simulations. D) Straight lines denote the moving average of the clonal (red) and twin spot (blue) shape distributions.

Next, we validated the overall distribution of cell shapes in the simulated tissue, i.e. the distribution of cells over the various possible polygon classes (i.e., number of edges per cell), and the dependency between polygon class and cell surface area. We based this on published experimental data (cell shape measurements) from refs [16] and [21]. This test further constrained our model parameters (Figure 5). As shown previously, the relative settings of the main parameters of the energy function (i.e., perimeter elasticity factor 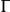 vs. line tension factor 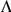) can be varied over a certain range, without resulting in much deviation between modeled and measured cell shapes. In our case, the added requirement of a stable boundary, which should mimic the actual boundary in the disc, constrained the parameters even further. For example, we noticed that relaxing the relative strength of the ‘perimeter elasticity’ parameter (third row in Figure 5) resulted in the best overall appearance of the boundary; however this was accompanied with a reduced fit to the polygon-distribution, and with somewhat unrealistic (elongated) cell shapes immediately adjacent to the boundary. As the best subjective compromise, we identified the parameter setting 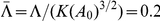 and 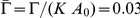 (first row in Figure 5). At this point in parameter space, we observed the best fit to known cell sizes and shapes, while at the same time obtaining a fairly straight boundary (see also the comparison to a negative control in Figure 3D). Immediately at the boundary, our model posits an approximately twelve-fold difference between TMx expression levels (anterior cells in row A1 having maximal TMx concentration of roughly 2.4 a.u. vs. posterior cells in row P1 with a basal TMx concentration of 0.2 a.u.; see Figures 3D and S1D). For average six-sided cells, this translates to a roughly two-fold increase in line tension at the boundary (see Figure S3F) – in good agreement with laser-ablation experiments [16], in which a 2.5 fold increase had been measured.

Our model qualitatively recapitulated the configurations observed in actual wing discs (including a localized boundary in spite of a longer-ranging response to Hh), so we next tested whether it would also correctly recapitulate the effects of genetic perturbations in the pathway. As described previously [20], [29], the transduction and response to the Hh signal can be blocked, in cells anterior to the boundary, by the removal of the essential Hh pathway protein Smo. This is achieved experimentally by inducing mitotic recombination in a small subset of cells, early in development, in larvae that are heterozygous for a mutant in the *smo* gene. The resulting small patches of homozygous mutant cells (clones) have been demonstrated to display two types of behavior [20], [29]: first, when situated close to the boundary, they tend to round off, minimizing their contact with neighboring cells. Second, when situated immediately adjacent to the boundary (specifically: at its anterior side), they tend to sort out from anterior cells and migrate into posterior territory. Both effects are interpreted as evidence for ectopic boundary formation – cells inside the clone are not receiving the Hh signal, but are juxtaposed to cells that do (this mimicks the situation at the boundary and leads to the rounding off, and/or to the migration into the posterior compartment that also does not transduce Hh). For our present study, we have repeated these experiments for a number of wing discs, and used automated image processing to quantify the extent of the “rounding-off” effect (Figure 4). We observed a highly significant distance-dependence of the rounding-off behavior: clones farther away from the boundary are rounding off less strongly than clones closer to the boundary (p = 6·10^−6^). As expected, this effect is not observed on the posterior side of the boundary, where Hh signaling has no known effects. This suggests that, whatever the molecular response to Hh signaling that is contributing to boundary formation, this response does extend further into the anterior tissue than just the immediate first row of cells at the boundary. In essence, cells seem to be “primed” for boundary formation, by Hh, several cell-diameters wide into the tissue.

In our model, we can arbitrarily set the Smo production rate to zero for any cell (and its descendents), thus mimicking the experimental situation. We find that we can qualitatively recapitulate the behavior of *smo^−^* clones in our simulations (Figure 4): clones situated close to the anterior side of the boundary, but not on the posterior side, can be observed to round off; in addition, we observe a tendency of clones that immediately straddle the boundary to migrate from anterior towards posterior territory (but not in the opposite direction). Importantly, similar to the experimental situation, we also observed a highly significant distance-dependence for the extent of rounding-off (with respect to the distance to the boundary, again only on the anterior side). This confirms that our model can correctly recapitulate this important aspect of the perturbation, and it supports our interpretation of the situation in the wing disc: a hypothetical transcriptional target of Hh signaling could be sufficient to generate a strictly local force that can establish a clearly delinated compartment boundary, despite this target being expressed (like all known transcriptional targets) over a certain distance away from the boundary. By assessing the shape of experimental *smo^−^* clones, we can effectively chart out the predicted expression level of this putative gene; it appears to be expressed roughly similar to *ptc* or *dpp* (in a graded stripe of expression along the boundary, at least 10 cell diameters wide).

When mutant cells are generated experimentally using mitotic recombination, a sister cell is generated that is not homozygous mutant, but instead homozygous wild-type in the *smo* gene. This so-called “twin-spot” provides another relevant input for our modeling: it presumably contains a larger amount of Smo protein (relative to the surrounding heterozygous tissue). We note that, both in the experiment and in our simulation, this difference in Smo levels does not suffice to generate a significant rounding-up of twin-spots (Figure 4). Indeed, the roundness of twin-spots is identical in the anterior and posterior compartment and independent from the distance towards the compartment boundary. Effectively, this observed behavior of experimental twin spots served as another constraint for our model parameterization: Differences in Hh pathway activity that are at most two-fold should not be sufficient to generate an observable boundary; and, the actual change in pathway activity at the endogenous boundary can thus be inferred to be much higher.

In an earlier version of the model, we had assumed that the amount of cortical constriction would simply be directly proportional to the hypothetical Hh target TMx (data not shown). However, under this assumption we were unable to find a parameter set that would satisfy all constraints and that would result in realistic cell shapes. Cells were either visibly too small or too large in the TMx expression stripe, and/or were showing imbalances in the relative contributions of cortical forces and area elasticity, leading to distorted cellular shapes (data not shown). In our view, this indicates that the processes at the boundary are not simply based on increasing or decreasing overall cortical constriction, but instead on a local redistribution of a pre-existing, basal propensity for cortical constriction. As an important consequence, it appears that it is not the absolute level of TMx that is important, but the *ratio* of TMx expression between two neighboring cells.

Our model is the first to couple tissue growth, driven by explicit cell divisions in a force-balanced cellular vertex approach, to signal transduction processes including diffusion, transcriptional responses and mechanical effects. This general approach should be applicable to a number of crucial developmental mechanisms, including growth control and body axis specification [24], [30], [31], [32]. In our case, we chose to model the Hh pathway, despite lacking many of the kinetic parameters that are needed to fully describe the pathway. This is probably the situation faced for most developmental signal transduction pathways today. However, we do believe this approach is justified, as long the as the outcome of the modeling is challenged experimentally, and as long as the sought-after answers are not addressing merely quantitative nuances in the pathway, but instead more fundamental mechanistic choices. Here, we essentially aimed to clarify whether a homotypic boundary model can work in principle (Figure 1), and whether a single, classical transcriptional target of Hh could be the missing link between pathway activity and physical forces at the cellular level. We find that this could indeed be the case, and that such a target gene might even be expressed at a basal level outside the Hh signaling stripe (since only relative differences at the boundary are needed). Our findings provide one possible explanation why previous attempts to search for this gene were unsuccessful: often it was assumed that the gene would be strongly expressed anteriorly, but not at all posteriorly. Instead, in our model the gene can indeed be expressed posteriorly (in fact, many configurations are possible, as long as they include a localized difference in expression at the boundary). Overall, our study indicates that mechanistic pathway modeling within whole tissues can help to choose among hypothetical, conflicting scenarios, and that it can even constrain properties of postulated missing players in a pathway.

## Material and Methods

### Fly stocks and genetics

To generate *smo* mutant clones, the *smo* ^3^ allele was flipped against a CD2-marked FRT chromosome. After mitotic recombination took place, non-CD2 expressing cells were homozygous mutant for *smo* ^3^. Cells of the posterior compartment were marked by expression of a *hh*-lacZ transgene. Flies had the following genotype: *y w* hsflp; FRT39 *smo* ^3^/FRT39 hs*CD2*; *hh*lacZ/+.

### Antibody staining

Antibody stainings of imaginal discs were done as described previously [33]. The following antibodies were used: rabbit α-E-Cadherin (Santa Cruz Biotechnology, 1∶200), mouse α-CD2 (Serotec, 1∶500), chicken α-βGal (Immunology Consultants Laboratory 1∶1000).

### Shape measurements of mutant clones

The shapes of *smo* ^−^ clones were determined by the absence of CD2 staining; correspondingly, the shapes of twin spots were defined by increased CD2 staining. The ‘roundness’ of *smo* ^−^ clones or twin spots was quantified by the measure 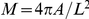 [34], where *A* is the area of the clone (or of the twin spot) and *L* its perimeter. Circular clones have 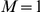, all other clones have 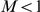. Clonal position was defined by the distance of the center of mass of the clone to the A/P boundary as marked by *hh-lacZ* staining. All geometry measurements in confocal microscopy images of wing discs, as well as in the corresponding images from simulations, were fully automatized with the help of the ImageProcessingToolbox*™* of Matlab.

### Modeling the Hedgehog pathway

We explicitly describe the Hedgehog pathway by a coupled system of ordinary and partial differential equations. The Hh protein, produced in the posterior compartment of the wing disc, diffuses into the anterior compartment, where it binds reversibly to its receptor Patched (Ptc). Binding of Hh to Ptc relieves the repression of the transmembrane protein Smoothened (Smo) by Ptc, but neither the mechanism for Ptc repression of Smo nor the mechanism by which the complex [Hh Ptch] relieves this repression has been fully understood. We assume that the active form of Smo corresponds to a complex of Smo protein and an unknown ligand Lx, [*Lx Smo*]. We further assume that, in a membrane compartment inaccessible to Smo, there exists a reservoir of Lx, from where it can flow towards Smo via a passive transport mechanism. We assume that Lx gets pumped away from Smo (active transport) by unbound Ptc. Ptc in turn is produced with a constant, low basal rate in the A-compartment, and is additionally a transcriptional target downstream of the active form of Smo in the A-compartment (via the transcription factor Ci, not modeled explicitly). Finally, we assume that the putative transmembrane protein “TMx” is likewise a transcriptional target downstream of the active form of Smo, with an additional, basal expression throughout the tissue. In Figure 3A, the above players and their interactions are summarized. This network of interactions is translated into a system of coupled ordinary and partial differential equations, listed in Figure 3B. Since cell-to-cell diffusion is restricted to the Hh molecule, only the first equation includes spatial derivatives, whereas all other equations are ordinary differential equations. We assume that protein kinetics can be described by a constant set of parameters for each protein [35]. *smo* ^−^ clones were mimicked by setting the corresponding production rate 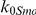 to zero; the corresponding twin spots were modeled by doubling this production rate as compared to wild type cells. The coefficients appearing in the system of equations are provided and described in the Supplemental Material (Text S1).

### A change in line tension leads to a straight boundary

The apical side of Drosophila's wing disc is modeled as a two-dimensional vertex model, where the junctions between cells are defined by straight lines (edges) connecting vertices. The resulting tissue topology is obtained by minimizing an energy function describing visco-elastic properties of the cells. Our model is an extension of previously published models describing cells as polygons [21], [26], [36]. In keeping with the framework of these previous models, mechanical forces are not stated explicitly; instead, by minimizing the ‘work function’ that aims to reflect the potential energy of the system, quasi-instantaneous relaxation of the system into local energy-minima is achieved [37]. This is assumed to correspond to the outcome of balanced forces acting in the elastic, dampened system of the tissue. It should be stressed that the modeling takes place on three, well-separated time scales: at the longest time scale (hours to days), cells divide and the tissue grows. At the medium time scale (minutes to hours), signaling proteins diffuse and are transduced into molecular responses inside the cell. The actual mechanics (forces and movements) occur at the shortest time scale – on the order of seconds – as has been demonstrated experimentally by tracking the relaxation movements following laser ablations in the tissue [16].

In our extension of the published models, we assume that a putative transmembrane protein downstream of the Hedgehog pathway (“TMx”) leads to a change in the line tension term of the energy function. We assume that TMx molecules are preferentially recruited to those edges that offer more binding partners (i.e., other TMx molecules expressed on neighboring cells). At the inner side of the cell membrane, TMx is assumed to signal to “effectors” that in turn influence cortical tension. The total number of effectors in each cell is not influenced by Hh signaling and is rate limiting. Both requirements are reflected in the definition of an additional scaling factor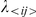 in the line tension contribution of the energy function displayed in Figure 3. Note that the sum in the definition of the scaling factor runs only over the edges of cell α.

The size of the scaling factor only depends on the *ratio* of the concentrations of two neighboring cells and is thus independent on the absolute values of concentrations (Figure S3). For all cells outside the stripe of increased TMx expression, the scaling factor computes to 1 and the energy function thus corresponds to the original energy function published in ref [21]. The scaling factor is strongly increased above the basal value of 1 on those edges of posterior cells immediately straddling the boundary (and thus touching anterior cells); and it is strongly decreased on all other edges of those cells. The changes in the scaling factor for anterior cells are more subtle, as shown in Figure S3. As each edge belongs to two cells, and their scaling factors for a given edge may not be the same, the energy function effectively takes into account the average of the two factors.

With the dimensionless parameters 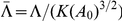 and 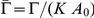, we obtain the following normalized energy function from the energy function displayed in panel C of Figure 3:
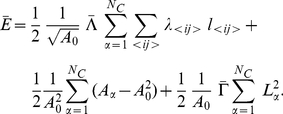
(1)We minimize the normalized energy function eq. (1) by a conjugate gradient method, leading to a shortening of edges that have an increased scaling factor and a lengthening of edges with a decreased scaling factor. This causes a straightening of the boundary between anterior and posterior cells, and (as an interesting side-effect) an increased average area of cells immediately posterior to the boundary (i.e., “P1” cells in Figure S2A; this has been experimentally observed as well [16]). Final simulations were run with the following parameter set for the normalized energy function: 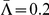, 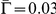 (and 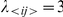 for edges of cells constituting the outer margin of the tissue). Assuming an average cell edge length of 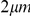, we applied the same target area to all cells, based on a regular hexagon with edge length *l*: 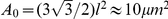.

### Modeling growth and the Hedgehog signaling pathway

The simulation of tissue growth was implemented as described in ref. [21]. In contrast to this previous work, we chose not to apply periodic boundary conditions, but modeled the tissue margins explicitly. Diffusion of Hh was discretized by the Finite Volume Method, using the cells as local control volumes. Following each growth step, the diffusion step was executed, and then the remaining of the differential equations displayed in Figure 3 (kinetic reactions) were solved numerically within each cell (for further details see Supplementary Information, Text S1). Simulations were started with 220 cells placed in a regular grid (always using the same starting formation). In simulations that included mutant clones, 20 *smo* ^−^ cells (simulated by a zero Smo production rate) adjacent to the corresponding twin spot cells (simulated by a doubled production rate of Smo as compared to wild type cells) were distributed uniformly in the starting configuration. We set the initial concentrations for all proteins within each cell to zero. Between cells and the extracellular medium we applied zero flux boundary conditions.

All simulations were run until the number of cells had increased to 6000; this roughly corresponds to the total number of cells in the pouch of a third-instar wing disc.

## Supporting Information

Figure S1 Simulated concentrations of major players of Hedgehog pathway. A)–D): Concentrations are displayed using Matlab's jet algorithm, where red corresponds to high, and blue to low concentrations. All concentrations are given in arbitrary units. B) Ptc_T_ is defined as the sum of free Ptc and ligand bound Ptc. E) Concentrations projected onto the anteroposterior axis. Note that the compartment boundary does not always remain precisely at the zero position, hence the slight ‘spread’ of the concentration curves (each cell in the tissue corresponds to one dot in the graphic).
(TIFF)

Figure S2Average apical cross-section areas of n-sided cells as a function of n. Areas are normalized to the average apical cross - section area 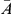 of each disc. Mean and standard error of the mean (SEM) are shown for 10 wing discs in both experiment and simulations. Note that the simulations reproduce the experimentally observed [16] increase in area of P1 cells. A) Data vs. simulation for all anterior (A), posterior (P), A1 and P1 cells. B) Data vs. simulation for all anterior (A), posterior (P), A2 and P2 cells.(TIFF)

Figure S3Converting TMx expression differences to localized edge constriction. Schematic representation of cells in the A2, A1, P1 and P2 rows of the wing pouch. A) Concentration of transmembrane protein TMx, per cell. All concentrations are expressed as multiples of the basal concentration 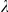 with 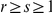. Only cells in the boundary region of the anterior compartment are exposed to concentrations of TMx higher than the basal concentration. The concentration is highest for A cells directly adjacent to the P compartment (“A1” cells) and decreases with the distance from boundary. B) We associate to each cell edge a term 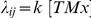 proportional to the TMx concentration of the cell it belongs to. For simplicity we have chosen 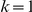. C) For neighboring edges with different values of 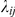, the higher value of both is given to both bonds (named in the following 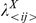). Together with the subsequence normalization, this mimics the fact that transmembrane proteins are preferentially recruited to edges offering more binding partners. D) Our assumption that the total line tension per cell is limited is modeled by normalizing 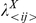 to the average value of 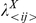 on all edges of a cell. Note that if all edges of a cell have the same value of 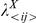, the scaling factor 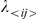 equals one for all edges of the cell. This is the case for all cells outside the stripe of increased expression of the transmembrane protein. E) Example for the calculation of the scaling factor with 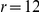 and 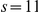 for regular hexagons. F) The effective average scaling factor 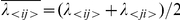 of each edge.(TIFF)

Figure S4 Dependency of boundary straightness on the ratio of TMx levels at the boundary. In this figure, the parameters chosen for the first panel of Figure 5 have been fixed, with the exception of *k^A^_LxSmo,2_* which has been varied to achieve different ratios of TMx between cells on either side of the boundary. TMx ratios of 6 and higher can be observed to result in a boundary quality approaching the actual situation in the wing disc.
(TIFF)

Video S1 Visualization of simulated dynamic boundary behavior. The movie shows a simulation run from 220 cells to roughly 4000 cells. The concentration of TMx is denoted by a white-to-purple color scale, where white denotes the basal, and purple the highest TMx concentration. In our model, all P-compartment cells express basal levels of TMx only. The TMx concentration alters the energy function as described in Figure 3. Note the stable separation of A and P compartment cells during growth.
(M4V)

Video S2 Control: TMx input into energy function is needed for boundary formation. The movie shows a simulation run from 220 cells to 6000 cells. Identical setup as in movie M1, but the TMx input to the energy function has been disabled. Note the mixing of cells at the compartment boundary, due to disturbances by random cell division events.
(M4V)

Video S3 Wing pouch with smo^−^ clones and corresponding twin spots. The movie shows a simulation run from 220 cells to 6000 cells. Wild type A-compartment cells are depicted in dark green, wild type P-compartment cells in blue. Cells mutant for the *smo* gene in the A-compartment are denoted in bright red, *smo^−^* cells in the P-compartment by a darker red. Twin spot cells are labeled by two shades of bright green, dependent on their compartment of origin. Note the migration of an anterior clone, originating adjacent to the compartment boundary, into the posterior compartment, as well as the rounding up of *smo^−^* clones in the boundary region of the anterior compartment.
(M4V)

Text S1 This text describes the modeling procedure in more detail, and lists parameters and initial conditions.
(PDF)
