# Supplementary figures and images for: Cell-Sorting at the A/P Boundary in the Drosophila Wing Primordium: A Computational Model to Consolidate Observed Non-Local Effects of Hh Signaling
Source: PLoS Comput Biol. 2011 Apr 7;7(4):e1002025. doi: 10.1371/journal.pcbi.1002025 (PMC3072364; doi:10.1371/journal.pcbi.1002025)

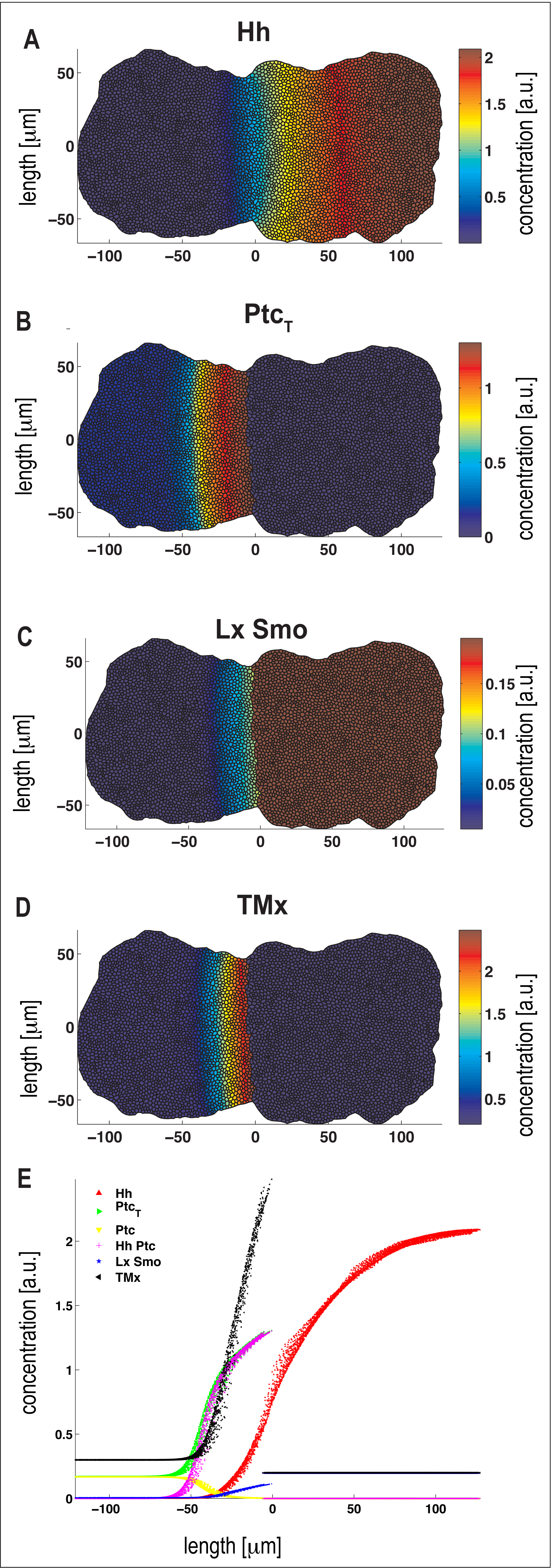

Supplement: Figure S1 — Simulated concentrations of major players of Hedgehog pathway. A)–D): Concentrations are displayed using Matlab's jet algorithm, where red corresponds to high, and blue to low concentrations. All concentrations are given in arbitrary units. B) PtcT is defined as the sum of free Ptc and ligand bound Ptc. E) Concentrations projected onto the anteroposterior axis. Note that the compartment boundary does not always remain precisely at the zero position, hence the slight ‘spread’ of the concentration curves (each cell in the tissue corresponds to one dot in the graphic). (TIFF) [file pcbi.1002025.s001.tif]

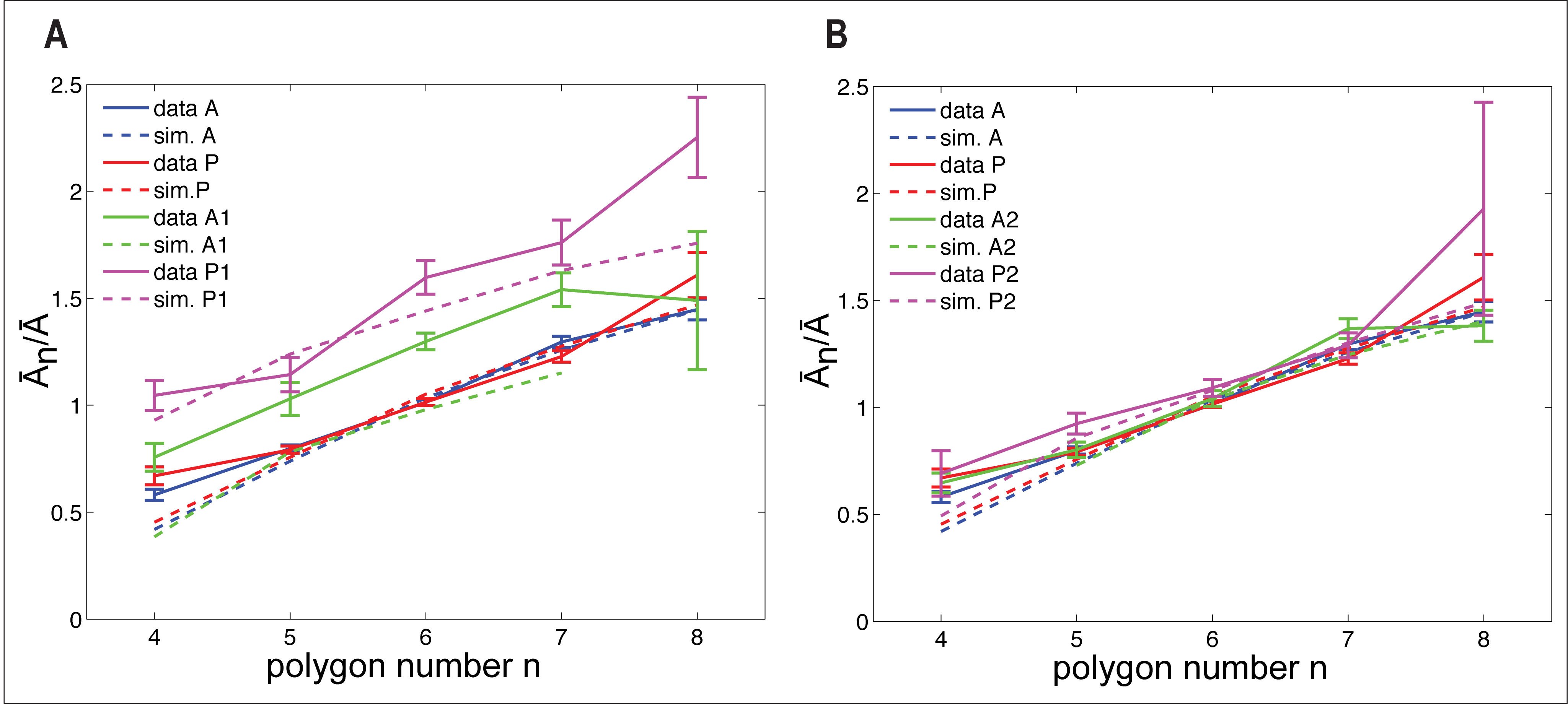

Supplement: Figure S2 — Average apical cross-section areas of n-sided cells as a function of n. Areas are normalized to the average apical cross - section area of each disc. Mean and standard error of the mean (SEM) are shown for 10 wing discs in both experiment and simulations. Note that the simulations reproduce the experimentally observed [16] increase in area of P1 cells. A) Data vs. simulation for all anterior (A), posterior (P), A1 and P1 cells. B) Data vs. simulation for all anterior (A), posterior (P), A2 and P2 cells. (TIFF) [file pcbi.1002025.s002.tif]

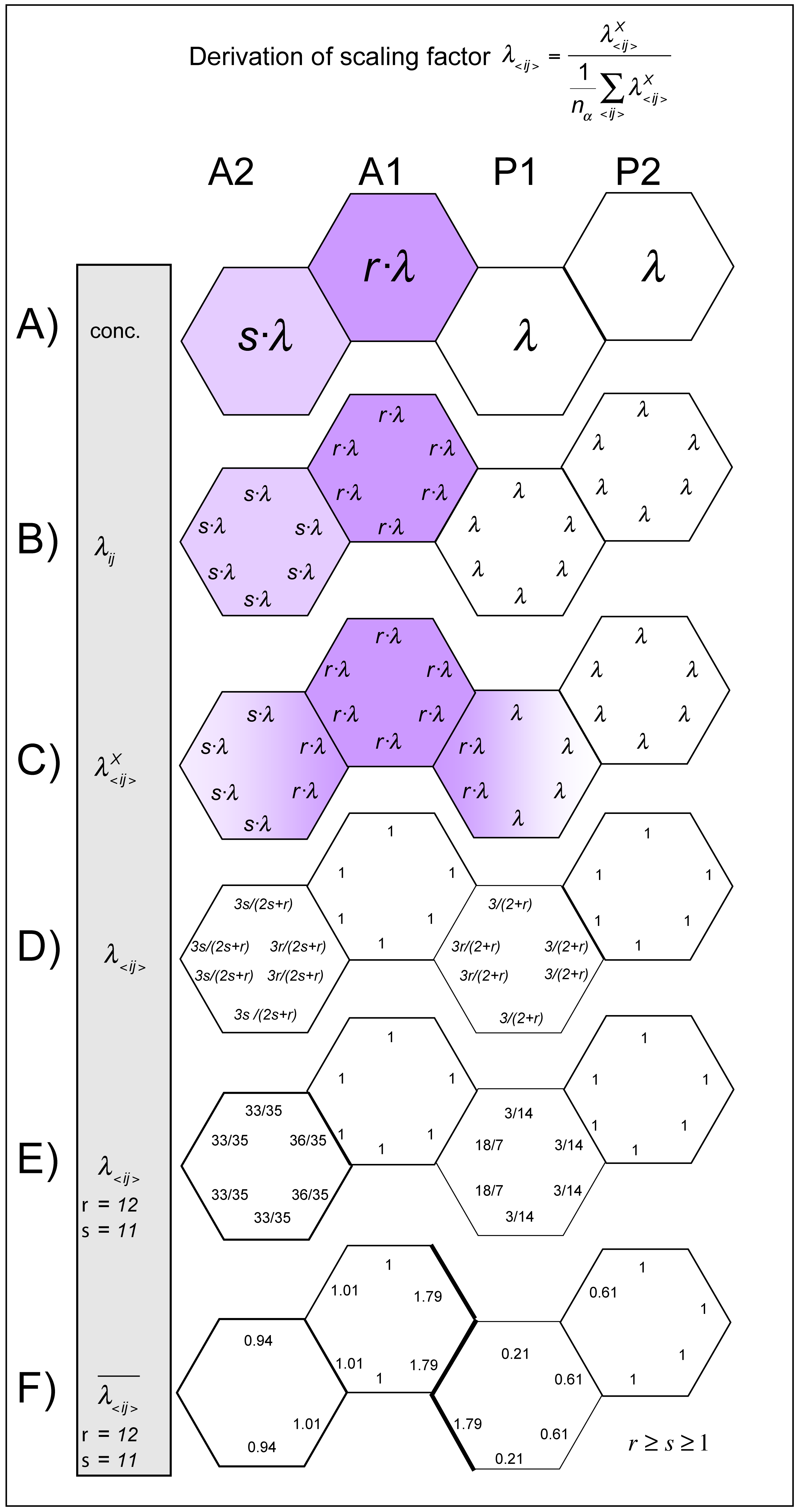

Supplement: Figure S3 — Converting TMx expression differences to localized edge constriction. Schematic representation of cells in the A2, A1, P1 and P2 rows of the wing pouch. A) Concentration of transmembrane protein TMx, per cell. All concentrations are expressed as multiples of the basal concentration with . Only cells in the boundary region of the anterior compartment are exposed to concentrations of TMx higher than the basal concentration. The concentration is highest for A cells directly adjacent to the P compartment (“A1” cells) and decreases with the distance from boundary. B) We associate to each cell edge a term proportional to the TMx concentration of the cell it belongs to. For simplicity we have chosen . C) For neighboring edges with different values of , the higher value of both is given to both bonds (named in the following ). Together with the subsequence normalization, this mimics the fact that transmembrane proteins are preferentially recruited to edges offering more binding partners. D) Our assumption that the total line tension per cell is limited is modeled by normalizing to the average value of on all edges of a cell. Note that if all edges of a cell have the same value of , the scaling factor equals one for all edges of the cell. This is the case for all cells outside the stripe of increased expression of the transmembrane protein. E) Example for the calculation of the scaling factor with and for regular hexagons. F) The effective average scaling factor of each edge. (TIFF) [file pcbi.1002025.s003.tif]

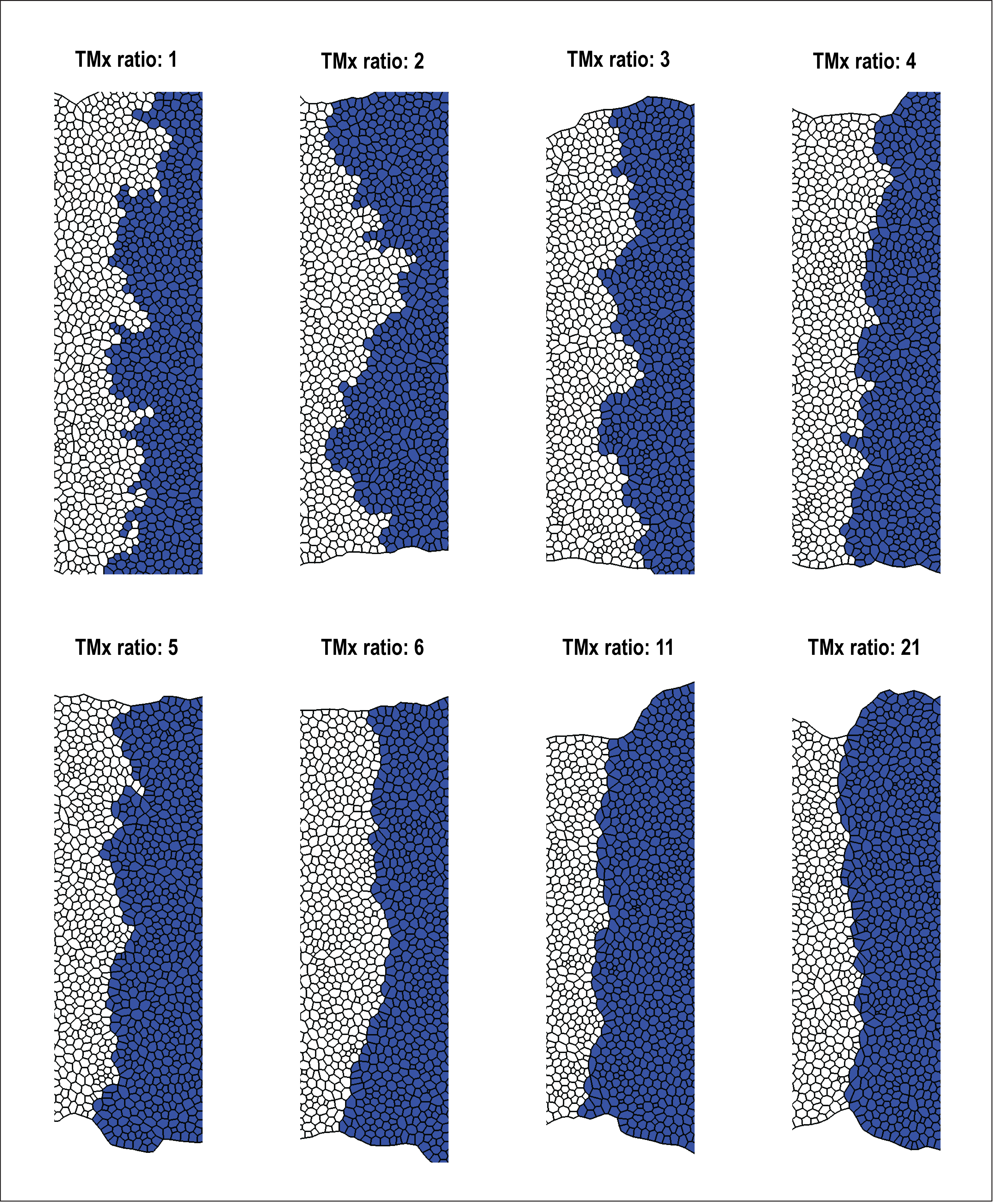

Supplement: Figure S4 — Dependency of boundary straightness on the ratio of TMx levels at the boundary. In this figure, the parameters chosen for the first panel of Figure 5 have been fixed, with the exception of kALxSmo,2 which has been varied to achieve different ratios of TMx between cells on either side of the boundary. TMx ratios of 6 and higher can be observed to result in a boundary quality approaching the actual situation in the wing disc. (TIFF) [file pcbi.1002025.s004.tif]
