# Supplementary material for: Cell-Sorting at the A/P Boundary in the Drosophila Wing Primordium: A Computational Model to Consolidate Observed Non-Local Effects of Hh Signaling
Source: PLoS Comput Biol. 2011 Apr 7;7(4):e1002025. doi: 10.1371/journal.pcbi.1002025 (PMC3072364; doi:10.1371/journal.pcbi.1002025)
Supplement: Text S1 — This text describes the modeling procedure in more detail, and lists parameters and initial conditions. (PDF) [file pcbi.1002025.s008.pdf]

Supplementary Information

Schilling et al.:

Cell-sorting at the A/P-compartment boundary in the Drosophila wing primordium: a computational model to consolidate observed non-local effects of Hedgehog signaling.

1 Discussion of Parameter Values

1.1 Parameter Set of the Differential Equations

For convenience we display again the system of differential equations describing the Hh signaling pathway:

$$\frac{\partial[Hh]}{\partial t} = D_{Hh}\nabla^2[Hh] - k_{Hh}\,[Hh] + k^P_{0\,Hh} - k^+_{Hh\cdot Ptc}[Hh][Ptc] + k^-_{HhPtc}[Hh\ Ptc], \quad \text{(S.1)}$$

$$\frac{\partial[Ptc]}{\partial t} = k^A_{0\,Ptc} + k^A_{LxSmo,1}\,[Lx\ Smo] - k_{Ptc} - k^+_{Hh\cdot Ptc}[Hh][Ptc] + k^-_{HhPtc}[Hh\ Ptc], \quad \text{(S.2)}$$

$$\frac{\partial[Hh\ Ptc]}{\partial t} = -k_{HhPtc}\,[Hh\ Ptc] + k^+_{Hh\cdot Ptc}[Hh][Ptc] - k^-_{HhPtc}[Hh\ Ptc], \quad \text{(S.3)}$$

$$\frac{\partial[Lx]}{\partial t} = -k_{Ptc\cdot Lx}\,[Ptc][Lx] + k_{Lx_{out}}\,[Lx_{out}] - k_{Lx}[Lx] - k^+_{Lx\cdot Smo}[Lx][Smo] + k^-_{LxSmo}[Lx\ Smo], \quad \text{(S.4)}$$

$$\frac{\partial[Smo]}{\partial t} = k_{0\,Smo} - k_{Smo}\,[Smo] - k^+_{Lx\cdot Smo}[Lx][Smo] + k^-_{LxSmo}[Lx\ Smo], \quad \text{(S.5)}$$

$$\frac{\partial[Lx\ Smo]}{\partial t} = -k_{LxSmo}\,[Lx\ Smo] + k^+_{Lx\cdot Smo}[Lx][Smo] - k^-_{LxSmo}[Lx\ Smo], \quad \text{(S.6)}$$

$$\frac{\partial[TMx]}{\partial t} = k_{0\,TMx} + k^A_{LxSmo,2}\,[Lx\ Smo] - k_{TMx}[TMx], \quad \text{(S.7)}$$

where

D_{Hh} : diffusion constant,

$[X]$: concentration of protein X,

$[L\ R]$: concentration of complex of ligand $[L]$ (Hh/Lx) and receptor $[R]$ (Ptc/Smo),

k^{sup}_{sub} : rate constants with

$sub = 0X$: zeroth, $sub = X$: first, $sub = X \cdot Y$: second order

$sup = A/P$: production in A/P compartment,

$sup = +/-$: reversible complex formation/degradation.

In Hh signaling, as in most other signaling pathways, quantitative data in terms of absolute molecular concentrations and rate constants is scarce. We therefore concentrated our parameter exploration on the better-known shapes (mostly based on antibody stainings) of the various concentration gradients. The parameter set displayed in Table (S.1) best describes known antibody staining data (from Suppl. Ref. [1]) as well as our own experimental observations.

We measured rounded smo^- clones in the anterior compartment (as an indicator for the priming of cells for boundary formation) up to 20 μm from the compartment boundary. Here we assume, that he priming of cells for boundary formation is due to the increased expression levels of the transmembrane protein TMx. Parameters were chosen such that the region of increased TMx expression roughly corresponds to the region of rounded smo^- clones (see Figure S1). Furthermore, the *relative* increase in TMx expression in the boundary region had to be large enough to obtain a straightened compartment

boundary (see Fig. (S4)) and smooth ectopic boundaries of smo^{-} clones in the boundary region.

Protein concentrations are given in arbitrary units (a.u.), as mostly data for ratios or normalized intensities of the various gradients is available. All variables are expressed as multiples of the characteristic length $L = 1\ \mu m$, the characteristic time $T = 1\ h$ and the characteristic concentration $U = 1\ a.u.$. With the help of these characteristic variables we transformed the system of partial and ordinary differential equations (S.1-S.7) into its non-dimensional form [2].

Table (S.1). Parameter set of the system of differential equations (S.1-S.7).

**Zero-Order Prod.**	$k_0 = 1$ a.u./h
$k^P_{0\ Hh}$	5 k_0
$k^A_{0\ Ptc}$	5 k_0
$k_{0\ Smo}$	5 k_0
$k_{0\ TMx}$	0.2 k_0
**First-Order Prod.**	$k_1 = 1$/h
$k^A_{Lx\ Smo,1}$	10 k_1
$k^A_{Lx\ Smo,2}$	20 k_1
$k_{Lx_{out}}$	k_1
**First-Order Decay**	
k_{Hh}	2 k_1
k_{Ptc}	30 k_1
k_{HhPtc}	5 k_1
k_{Lx}	3 k_1
k_{Smo}	10 k_1
k_{LxSmo}	10 k_1
k_{TMx}	k_1
k^-_{HhPtc}	k_1
k^-_{LxSmo}	k_1
**Second-Order Prod.**	$k_2 = 1/((\text{a.u.}) \cdot \text{h})$
$k_{Ptc \cdot Lx}$	$5 \cdot 10^4\ k_2$
$k^+_{Hh \cdot Ptc}$	600 k_2
$k^+_{Smo \cdot Lx}$	60 k_2
**Concentrations**	
Lx_{out}	2 a.u.
**Diffusion Constant**	
D_{Hh}	$2 \cdot 10^{-13}\ m^2/s = 720\ \mu m^2/h$

The chosen value for the Hh diffusion constant, $D_{Hh} = 2 \cdot 10^{-13}\ m^2/s$, is is in the same order of magnitude as the measured effective diffusion coefficient of GFP-Dpp: $D_{Dpp} = (1 \pm 0.5) \cdot 10^{-13} m^2/s$ [3]. Note that both diffusion constants are about three orders of magnitude smaller than typical aqueous diffusion coefficients of proteins in free solution. However, the diffusion term in eq. (S.1) represents the mass exchange of Hh between (and not within) cells, which is presumably not a free aqueous diffusion process.

1.2 Parameter Exploration of the Energy function

We explored the parameter space of the constants entering the normalized energy function, $\overline{\Lambda}$ and $\overline{\Gamma}$, in steps of 0.01 for both parameters (simulations not shown). The goal was to find a parameter set in agreement with our experimental data and with data describing general tissue topology properties. In general, stable parameter sets (which allowed to simulate growth) with a higher ratio of $\overline{\Lambda}$ to $\overline{\Gamma}$ resulted in straighter compartment boundaries and rounder smo^{-}clones in the region of enhanced TMx expression (Figure 5). In these regions of parameter space, the relative contribution of the line tension term to the total energy of the tissue is enhanced and thus changes in the line tension contribution due to Hh signaling have a stronger impact. The chosen parameter set of $\overline{\Lambda} = 0.2$ and $\overline{\Gamma} = 0.03$ is in agreement with our experimental findings and with data published in [4, 5].

1.3 Parameter Exploration of the Kinetic Parameters

In our model, the sole interface between Hedgehog pathway activity and mechanical outcome in the tissue is the "TMx"-molecule. More specifically, the relative difference of TMx levels between two cells influences the contraction properties of the edge-interface between the two cells. Below we explore this difference systematically, assuming steady-state conditions. For the concentration of the transmembrane protein we have (see Fig. (3) and eqn. (S.7))

$$\frac{\partial[TMx]}{\partial t} = k_{0\ TMx} + k^{A}_{LxSmo,2}\ [Lx\ Smo] - k_{TMx}[TMx]. \tag{S.8}$$

Hedghog target genes are only present in the anterior compartment, thus we assume that the active form of Smoothened, $[Lx\ Smo]$, leads to an (enhanced) expression of the Hedgehog target genes only in the anterior compartment. This is reflected in the definition of the production rate $k^{A}_{LxSmo,2}$, where the superscript A denotes a production rate only present in the anterior compartment. The concentration of the active form of Smoothened, $[Lx\ Smo]$, is approximately zero (see panel C of figure S1) outside the Hedgehog stripe in the anterior compartment. Thus we obtain from eq. (S.8) for the basal concentration of transmembrane protein outside the Hedgehog stripe:

$$\frac{\partial[TMx]_{basal}}{\partial t} = k_{0\ TMx} - k_{TMx}[TMx]_{basal}. \tag{S.9}$$

For the steady state basal TMx concentration we get from eq. (S.9)

$$\begin{aligned} 0 &= k_{0\ TMx} - k_{TMx}[TMx]_{basal} \Rightarrow \\ [TMx]_{basal} &= k_{0\ TMx}/k_{TMx}. \end{aligned} \tag{S.10}$$

In the region of enhanced concentration of the active form of Smoothened we get from eq. (S.8) in steady state

$$\begin{aligned} [TMx] &= k_{0\ TMx}/k_{TMx} + k^{A}_{LxSmo,2}/k_{TMx}[Lx\ Smo] \\ &= [TMx]_{basal} + k^{A}_{LxSmo,2}/k_{TMx}[Lx\ Smo]. \end{aligned} \tag{S.11}$$

The highest concentration difference between neighboring cells is between P1 (basal concentration of transmembrane protein TMx_{basal}) and A1 (highest concentration TMx^{A1}_{max}) cells (see Figure 3D), their shared edges define the compartment boundary between posterior and anterior compartment.

For the ratio of transmembrane concentrations between a A1 and P1 we have

$$r = \frac{[TMx]^{A1}_{max}}{[TMx]_{basal}} = 1 + k^{A}_{LxSmo,2}/k_{0\ TMx}[Lx\ Smo]^{A1}_{max}. \tag{S.12}$$

$[Lx\ Smo]^{A1}_{max}$ is the maximal concentration of $[Lx\ Smo]$ in the anterior compartment. By varying $k^{A}_{LxSmo,2}$ we explored in Fig. (S.4) the dependency of boundary straightness on the ratio r of $[TMx]$ levels on either side of the boundary.

2 Modeling Growth and the Hh Signaling Pathway

All simulations were started with 220 cells; the initial concentrations of all proteins within each cell was set to zero. Between cells and the extracellular medium we applied zero flux boundary conditions. Mimicking the relatively quiescent first instar stage of the larvae, the system of differential equations (S.1-S.7) was solved without cell divisions within the first 20 h simulated time. From $t = 20\ h$ on, we considered cell devision and energy minimization after each simulated minute, whereas the system of differential equations was simulated with a temporal resolution of $\Delta t = 1\ s$. Simulations were stopped, when the tissue was grown up to 6000 cells. This roughly corresponds to the number of cells in the wing pouch of a third instar larva.

3 Numerical Solution of the System of Differential Equations

The Operator Split approach (OS) applied in this study is based on the assumption that chemical reactions and cell-to-cell transport by diffusion occur on different time scales, thus transport and chemical reaction processes can be decoupled. Diffusion and reaction operators are separated and solved sequentially in each time step Δt [6, 7].

3.1 Langmuir Assumptions

The kinetics of the reversible binding of a ligand L (here: Hh/Ptc) to its (immobile) receptor R (here: Lx/Smo) is given by

$$\frac{\partial [LR]}{\partial t}\bigg\|_{reac} = k^{+}_{L\ R}[L][R] - k^{-}_{L\ R}[LR]. \tag{S.13}$$

In local equilibrium of binding of receptor to its ligand, we obtain

$$\frac{\partial [LR]}{\partial t}\bigg\|_{reac} = 0 \Rightarrow \frac{[LR]}{[L][R]} = \frac{k^{+}_{LR}}{k^{-}_{LR}} = K_{LR}. \tag{S.14}$$

The total amount of receptor is given by the sum of free and complex bound receptor

$$[R_T] = [R] + [LR]. \tag{S.15}$$

With eq. (S.14) we get for the relation of complex to total receptor in the local equilibrium

$$[LR] = \Theta R_T, \tag{S.16}$$

where

$$\Theta = \frac{K_{LR}[L]}{1 + K_{LR}[L]} \tag{S.17}$$

is the Langmuir equation [8]. It relates the adsorption of molecules onto a limited number of binding sites to the concentration of $[L]$ in the solution at a fixed temperature. Θ is the relative coverage of the binding sites. The bigger K_{LR}, the closer is Θ to its asymptotical value of 1.

3.2 Algorithm

Below, $[X]_n$ denotes the concentration obtained in a previous time step, $[X]^*_{n+1}$ is an intermediate concentration, and $[X]_{n+1}$ is the concentration at the end of the current time step. In each time step, the following algorithm is executed:

1. Computation of the concentration of complex in local equilibrium $[LR]_n$ from $[L]_n$ by eq. (S.16)

$$[LR]_n = \Theta_n [R]_{T,n} = \frac{K_{LR}[L]_n}{1 + K_{LR}[L]_n}. \tag{S.18}$$

2. Calculation of intermediate concentration of ligands Hh and Lx

   - Ligand Hedgehog:
     We assume that cell-to-cell diffusion occurs on a different time-scale than ligand receptor binding and unbinding. This allows us to simulate the diffusion and zero-order production and first-order decay of Hedgehog without changing the receptor-bound concentration $[Hh\ Ptc]$. Thus we obtain from eq. (S.1)

$$\left(\frac{1}{\Delta t} + k_{Hh} - D_{Hh}\nabla^2\right)[Hh]^*_{n+1} = \frac{1}{\Delta\ t}[Hh]_n + k^0_{Hh}. \tag{S.19}$$

     in which $[Hh]^*_{n+1}$ is the intermediate concentration of Hh within each cell. Eq. (S.19) is discretized in space by the Finite Volume Method (FVM) [9], in which the domain is divided into a number of control volumes, here polygons defined by the vertices of tissue cells. The differential form of the governing equation is integrated over each control volume resulting in a set of ordinary differential equations for cell-averaged concentrations. By construction, the FVM conserves mass in each individual cell.

   - Lipid like ligand Lx
     Assuming local equilibrium of complex $[LxSmo]$ and no change in Ptc concentration we obtain by integration:

$$[Lx]^*_{n+1} = \frac{e^{-\Delta t y}\left(-k_{Lx_{out}}[Lx_{out}] + k_{Lx}[Lx]_n + k_{Pt\cdot Lx}[Lx]_n[Pt]_n\right) + k_{Lx_{out}}[Lx_{out}]}{y}, \tag{S.20}$$

     where

$$y = k_{Lx} + k_{Ptc\cdot Lx}[Pt]_n.$$

3. Evaluation of the total concentration of the ligand

$$[L]^*_{T,n+1} = [L]^*_{n+1} + [LR]_n. \tag{S.21}$$

4. Calculation of the free ligand concentration $[L]_{n+1}$ from the total concentration $[L]^*_{T,n+1}$
   Substituting the Langmuir equation into the definition of the total concentration $[L]^*_{T,n+1}$ results in:

$$[L]^*_{T,n+1} = [L]_{n+1} + \frac{K_{LR}[L]_{n+1}}{1 + K_{LR}[L]_{n+1}}[R]_{T,n}. \tag{S.22}$$

   Solving the above equation for $[L]_{n+1}$, we obtain:

$$[L]_{n+1} = \frac{a + \sqrt{a^2 + 4K_{LR}\ [L]^*_{T,n+1}}}{2K_{LR}}, \tag{S.23}$$

   where

$$a = [L]^*_{T,n+1}K_{LR} - 1 - K_{LR}[R]_{T,n}.$$

5. Calculation of intermediate values of the complexes $[LR]^*_{n+1}$ and receptors$[R]^*_{n+1}$
   From eq. (S.22) follows

$$[LR]^*_{n+1} = [L]^*_{T,n+1} - [L]_{n+1}, \tag{S.24}$$

$$[R]^*_{n+1} = [R]_{T,n} - [LR]^*_{n+1}. \tag{S.25}$$

6. Kinetic reactions
   Considering relatively slow concentration changes of reaction parterns, we solve the pseudo first-order approximations of all kinetic reaction equations (except for those related to Hh production and decay) analytically, resulting in exponential expressions:

   (a) Complexes

$$[LR]_{n+1} = [LR]^*_{n+1} \exp\left(-\Delta t\, k_{LR}\right). \tag{S.26}$$

   (b) Receptor Patched

$$[Ptc]_{n+1} = \left(\frac{k^A_{0\ Ptc}}{k_{Ptc}} + \frac{k^A_{Lx\ Smo,1}}{k_{Ptc}}[Lx\ Smo]_{n+1}\right)\left(1 - e^{-k_{Ptc}\Delta t}\right) + e^{-k_{Ptc}\Delta t}[Ptc]^*_{n+1} \tag{S.27}$$

   (c) Receptor Smoothened

$$[Smo]_{n+1} = \frac{k^0_{Smo}}{k_{Smo}}\left(1 - e^{-k_{Smo}\Delta t}\right) + e^{-k_{Smo}\Delta t}[Smo]_{n+1}. \tag{S.28}$$

   (d) Transmembrane protein

$$\begin{aligned}[TMx]_{n+1} = &\left(\frac{k^A_{0\ TMx}}{k_{TMx}} + \frac{k^A_{Lx\ Smo,2}}{k_{TMx}}[Lx\ Smo]_{n+1}\right)\left(1 - e^{-k_{TMx}\Delta t}\right)\\ &+ e^{-k_{TMx}\Delta t}[TMx]^*_{n+1}\end{aligned} \tag{S.29}$$

7. Equilibration of complexes and receptors

$$[L]_{T,n+1} = [L]_{n+1} + [LR]_{n+1}, \tag{S.30}$$

$$[R]_{T,n+1} = [R]_{n+1} + [LR]_{n+1}. \tag{S.31}$$

8. Repetition of entire procedure for each time step

References

1. Casali A, Struhl G (2004) Reading the hedgehog morphogen gradient by measuring the ratio of bound to unbound patched protein. Nature 431: 76–80.

2. Dillon R, Othmer HG (1999) A mathematical model for outgrowth and spatial patterning of the vertebrate limb bud. Journal of Theoretical Biology 197: 295–330.

3. Kicheva A, Pantazis P, Bollenbach T, Kalaidzidis Y, Bittig T, et al. (2007) Kinetics of morphogen gradient formation. Science 315: 521–5.

4. Farhadifar R, Röper JC, Aigouy B, Eaton S, Jülicher F (2007) The influence of cell mechanics, cell-cell interactions, and proliferation on epithelial packing. Curr Biol 17: 2095–104.

5. Landsberg KP, Farhadifar R, Ranft J, Umetsu D, Widmann TJ, et al. (2009) Increased cell bond tension governs cell sorting at the drosophila anteroposterior compartment boundary. Curr Biol 19: 1950–5.

6. Yeh G, Tripathi V (1989) A critical-evaluation of recent developments in hydrogeochemical transport models of reactive multichemical components. Water Resour Res 25: 93–108.

7. Fahs M, Carrayrou J, Younes A, Ackerer P (2008) On the efficiency of the direct substitution approach for reactive transport problems in porous media. Water Air Soil Poll 193: 299–308.

8. Langmuir I (1916) The constitution and fundamental properties of solids and liquids. J Am Chem Soc 38: 2221–2295.

9. Versteeg HK, Malalasekera W (2007) An introduction to computational fluid dynamics: the finite volume method : 503.
